# Gene expression profiles for an immunoscore model in bone and soft tissue sarcoma

**DOI:** 10.18632/aging.202956

**Published:** 2021-05-04

**Authors:** Jingyuan Fan, Xinyi Qin, Rongquan He, Jie Ma, Qingjun Wei

**Affiliations:** 1 Department of Orthopedics, The First Affiliated Hospital of Guangxi Medical University, Nanning, Guangxi, China; 2 School of Graduate, Guangxi Medical University, Nanning, Guangxi, China; 3 Department of Oncology, The First Affiliated Hospital of Guangxi Medical University, Nanning, Guangxi, China

**Keywords:** immune infiltrating, sarcoma, CIBERSORT, K-means, lasso regression

## Abstract

Background: Immune infiltration is a prognostic marker to clinical outcomes in various solid tumors. However, reports that focus on bone and soft tissue sarcoma are rare. The study aimed to analyze and identify how immune components influence prognosis and develop a novel prognostic system for sarcomas.

Methods: We retrieved the gene expression data from 3 online databases (GEO, TCGA, and TARGET). The immune fraction was estimated using the CIBERSORT algorithm. After that, we re-clustered samples by K-means and constructed immunoscore by the least absolute shrinkage and selection operator (LASSO) Cox regression model. Next, to confirm the prognostic value, nomograms were constructed.

Results: 334 samples diagnosed with 8 tumor types (including osteosarcoma) were involved in our analysis. Patients were next re-clustered into three subgroups (OS, SAR1, and SAR2) through immune composition. Survival analysis showed a significant difference between the two soft tissue groups: patients with a higher proportion of CD8+ T cells, macrophages M1, and mast cells had favorable outcomes (p=0.0018). Immunoscore models were successfully established in OS and SAR2 groups consisting of 12 and 9 cell fractions, respectively. We found immunosocre was an independent factor for overall survival time. Patients with higher immunoscore had poor prognosis (p<0.0001). Patients with metastatic lesions scored higher than those counterparts with localized tumors (p<0.05).

Conclusions: Immune fractions could be a useful tool for the classification and prognosis of bone and soft tissue sarcoma patients. This proposed immunoscore showed a promising impact on survival prediction.

## INTRODUCTION

Bone and soft-tissue sarcomas are a family of malignant tumors that are arisen from mesenchymal and connective tissue cells, accounting for about 1% of the total reported malignancies in 2018 [1]. Due to the heterogeneity, the patient’s prognosis is highly variable. Several prediction models were investigated and used to predict disease progression and overall survival [2–4]. Various prognostic factors such as age, tumor size, depth, surgical margin, histological subtype, site, vascular invasion, and adjuvant chemo- and/or radiotherapy [3–8] have been shown to have a compelling impact on survival. However, a common weakness of current models is that the sarcoma subgroup was identified by an extensive histological subclassification and had been continuously refined during the last few years. Therefore, those prediction models could sometimes be unstable due to the changeable histologic subtypes. Thus, to predict patient outcomes more reliably and precisely, a prediction model with new factors is needed.

Recently, increasing evidence indicated that immune cells may have some complex interactions with tumor cells within the tumor microenvironment [9], which are associated with immunotherapy response in patients with advanced tumors. Although the composition of the immune microenvironment is heterogeneous according to the type of cancer and stage, it is well established that some specific patterns of immune infiltration are correlated with preferable outcomes [10–12]. For instance, CD8+ T cell has the capacity of killing tumor cells; thus, the infiltration of CD8+ T cell often indicates a better outcome [13]. In contrast, several immune cells, such as regulatory T-cells (Treg), can promote immune evasion and associate with poor prognosis [14].

To increase prediction accuracy, an immune scoring system based on the type, density, and location of immune infiltration was proposed [15]. Also, this prognostic factor’s clinical value was repeatedly validated in the past few years among a variety of tumors [15–17], including bone and soft tissue sarcomas [13, 18, 19]. Therefore, it may help clinicians predict patient outcomes by incorporating the survival effect of immune infiltration into the current staging system and prediction models.

Numerous cells characterize the immune infiltration, and their prognostic impact differs in different kinds of cancer [20]. CIBERSORT is a developed computational method to characterize complex tissues’ cell composition through the gene expression profiles [21]. LM22 is a gene file comprising 547 genes, designed to distinguish 22 mature human hematopoietic populations and activation states [21]. In light of this, we applied the CIBERSORT algorithm with LM22 to enumerate the proportions of immune cell types on gene expression data, the K-means algorithm, to regroup samples. Then, least absolute shrinkage and selection operator (LASSO) Cox regression analysis was performed to calculate the immunoscores of each patient, trying to discuss the prediction value of immunoscore in bone and soft-tissue sarcoma.

To the best of our knowledge, this is the first construction of immunoscore model in patients with bone and soft-tissue sarcoma and the immune study with the largest sample size. We believe our results could provide new directions for the prognostic researches of bone and soft-tissue sarcoma.

## RESULTS

**Figure 1:**
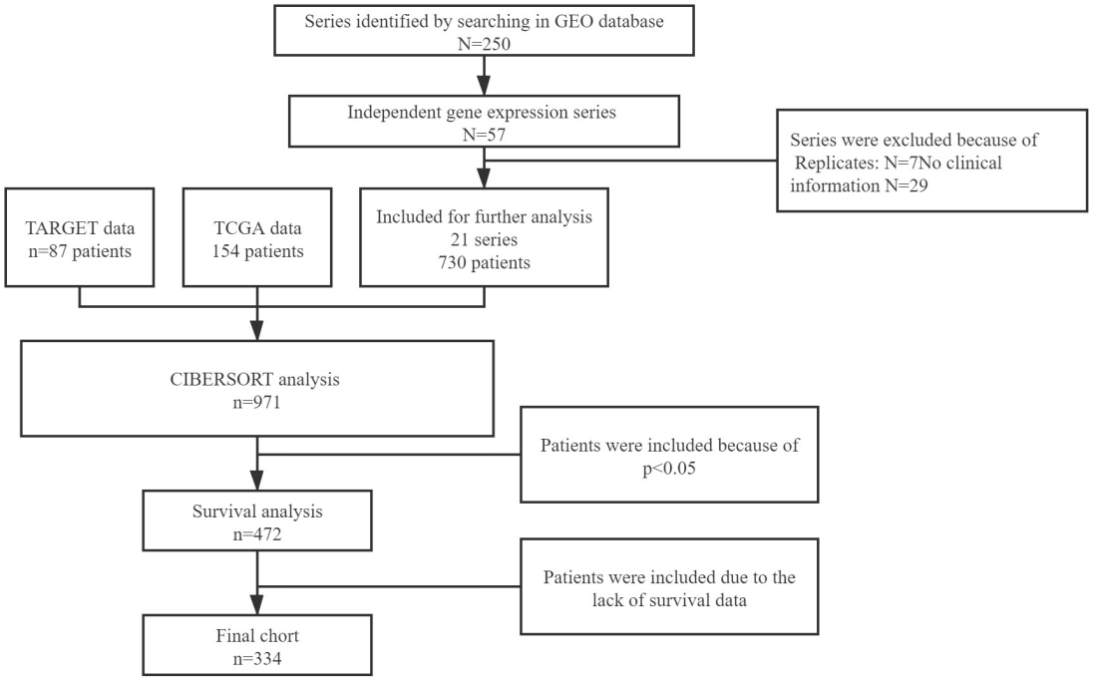
Flow chart of data collection.

After the searching and downloading online data, 472 samples diagnosed with 8 different sarcoma types were involved in our survival analysis. Figure 1 showed the process of selection. And then, 138 samples were excluded due to the overall survival information was not available. The general characteristics of 334 included patients are shown in Table 1. Of these samples, 123 cases were diagnosed with osteosarcoma, and 211 were soft tissue sarcoma. Soft tissue sarcomas were further divided into 7 types, including Ewing sarcoma (ES), fibrosarcoma (FBS), leiomyosarcoma (LMS), liposarcoma (LPS), synovial sarcoma (SS), undifferentiated pleomorphic sarcoma (UPS), and others (not classified). Several covariates, such as tumor size and tumor stage, were failed to enter the uni- or multivariable model because of the insufficient number of cases. Ultimately, age, sex, tumor type, tumor site, and immune composition were included in the subsequent analysis.

**Table 1:** Basic patient characteristics.

**Items**	**Whole (n=334)**	**OS (n=123)**	**SAR1 (n=78)**	**SAR2 (N=133)**	**p**
Age					
<18y	130(38.92%)	90(73.17%)	35(44.87%)	5(3.76%)	<0.001
≥18y	204(61.08%)	33(26.83%)	43(55.13%)	128(96.24%)	
Sex					
Male	191(57.19%)	74(60.16%)	51(65.38%)	66(49.62%)	0.058
Female	143(42.81%)	49(39.84%)	27(34.62%)	67(50.38%)	
Tumor type					
Ewing sarcoma (ES)	49(14.67%)	0(0.00%)	39(50.00%)	10(7.52%)	<0.001
Fibrosarcoma (FBS)	20(5.99%)	0(0.00%)	3(3.85%)	17(12.78%)	
Leiomyosarcoma (LMS)	51(15.27%)	0(0.00%)	11(14.10%)	40(30.08%)	
Liposarcoma (LPS)	36(10.78%)	0(0.00%)	5(6.41%)	31(23.31%)	
Osteosarcoma (OS)	123(36.83%)	123(100.00%)	0(0.00%)	0(0.00%)	
Synovial sarcoma (SS)	1(0.30%)	0(0.00%)	0(0.00%)	1(0.75%)	
Undifferentiated pleomorphic sarcoma (UPS)	41(12.28%)	0(0.00%)	12(15.38%)	29(21.80%)	
Others	13(3.89%)	0(0.00%)	8(10.26%)	5(3.76%)	
Tumor site					
lower limbs	155(46.41%)	108(87.80%)	9(11.54%)	38(28.57%)	<0.001
upper limbs	18(5.39%)	12(9.76%)	3(3.85%)	3(2.26%)	
trunk	25(7.49%)	3(2.43%)	6(7.69%)	16(12.03%)	
Unknown	136(40.60%)	0(0.00%)	60(76.92%)	76(57.14%)	
Tumor size					
<5cm	21(6.29%)	0(0.00%)	3(3.84%)	18(13.53%)	<0.001
≥5cm	118(35.33%)	0(0.00%)	25(32.05%)	93(69.92%)	
Unknown	195(58.38%)	123(100.00%)	50(64.10%)	22(16.54%)	
Adjuvant chemotherapy					
Yes	151(45.21%)	52(42.28%)	20(25.64%)	79(59.40%)	<0.001
No	20(5.99%)	0(0.00%)	3(3.85%)	17(12.78%)	
Unknown	163(48.80%)	71(57.72%)	55(70.51%)	37(27.82%)	
ovs	49.89±42.68	57.79±47.74	53.78±43.54	40.30±34.98	0.0021
3-year survival rate	50.30%	56.91%	56.41%	40.60%	0.016
5-year survival rate	31.14%	37.40%	34.62%	23.31%	0.039

### The distinction of immune component and overall survival time

**Figure 2:**
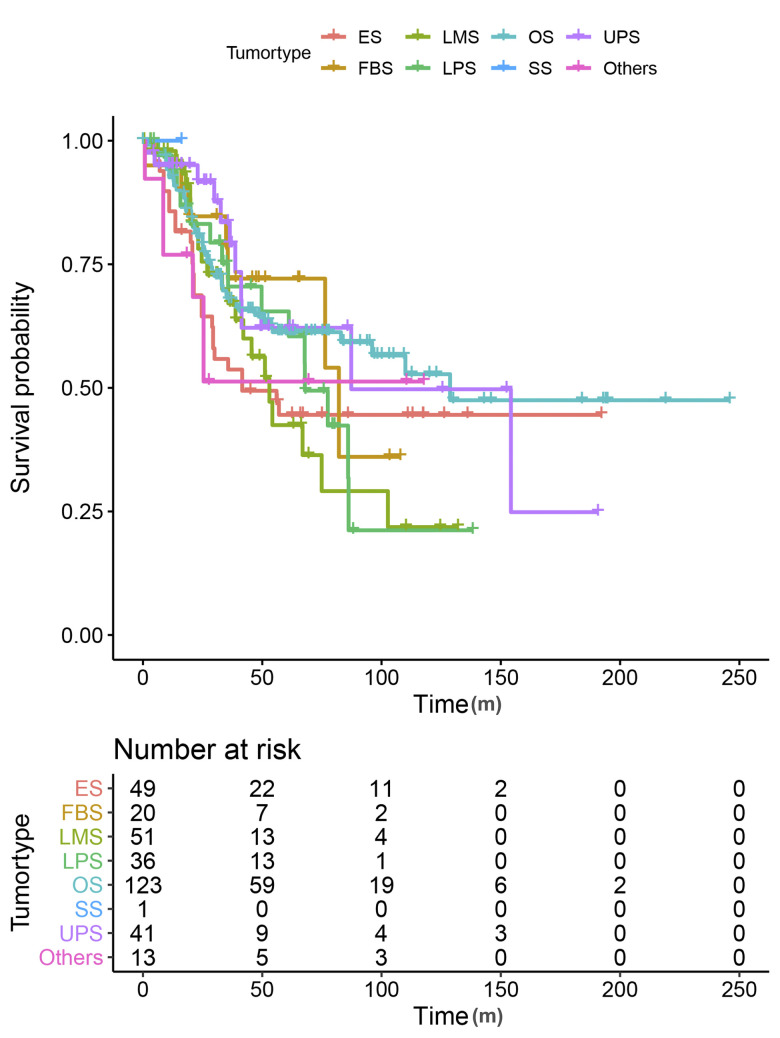
Kaplan-Meier curves for overall survival by tumor types.

**Figure 3:**
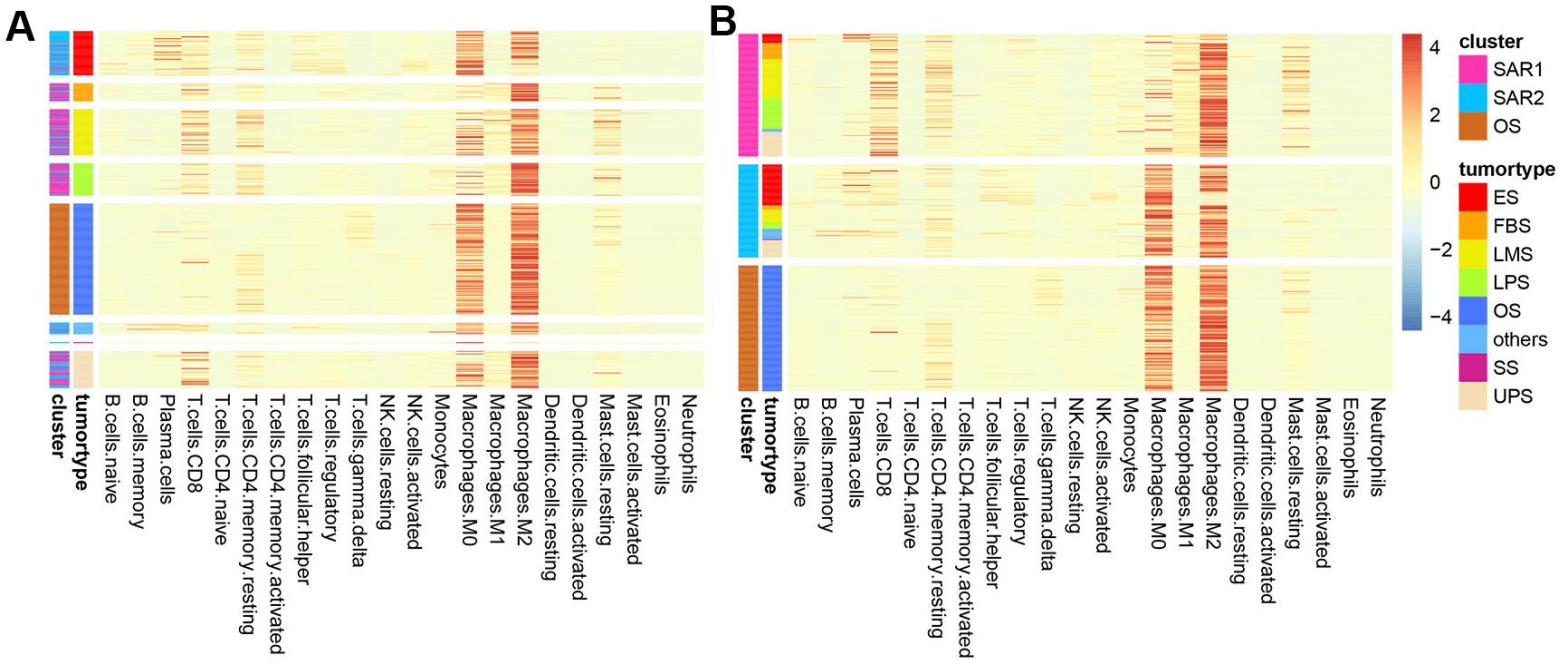
**Heatmap of immune composition.** (**A**) Samples were clustered by tumor types; (**B**) samples were clustered by the results of K-means.

Figure 2 and Table 1 indicated an obvious distinction in survival time among different types of sarcoma. The median survival time varied from 41.6 months (Ewing sarcoma) to 128.7 months (osteosarcoma). Then the heatmap was conducted based on LM22 signatures, which provided a summary of the immune cell composition across sarcoma types (Figure 3A). The 5 most common immune cell fractions were M2 macrophages (33.89%), M0 macrophages (21.33%), memory resting CD4+ T cells. (7.24%), CD8+ T cells. (7.11%) and resting mast cells (5.76%). However, the characteristics of immune cell composition in each sarcoma type are not completely the same. For instance, fibrosarcoma, leiomyosarcoma, and liposarcoma patients have a higher fraction of M0 macrophages and lower resting mast cells than other cases. Besides, the composition was slightly different, even within the same tumor type. We assumed the fraction of immune cells may be relevant to tumor prognosis and regrouped cases according to the immune components.

### New sarcoma classification through immune composition

**Figure 4:**
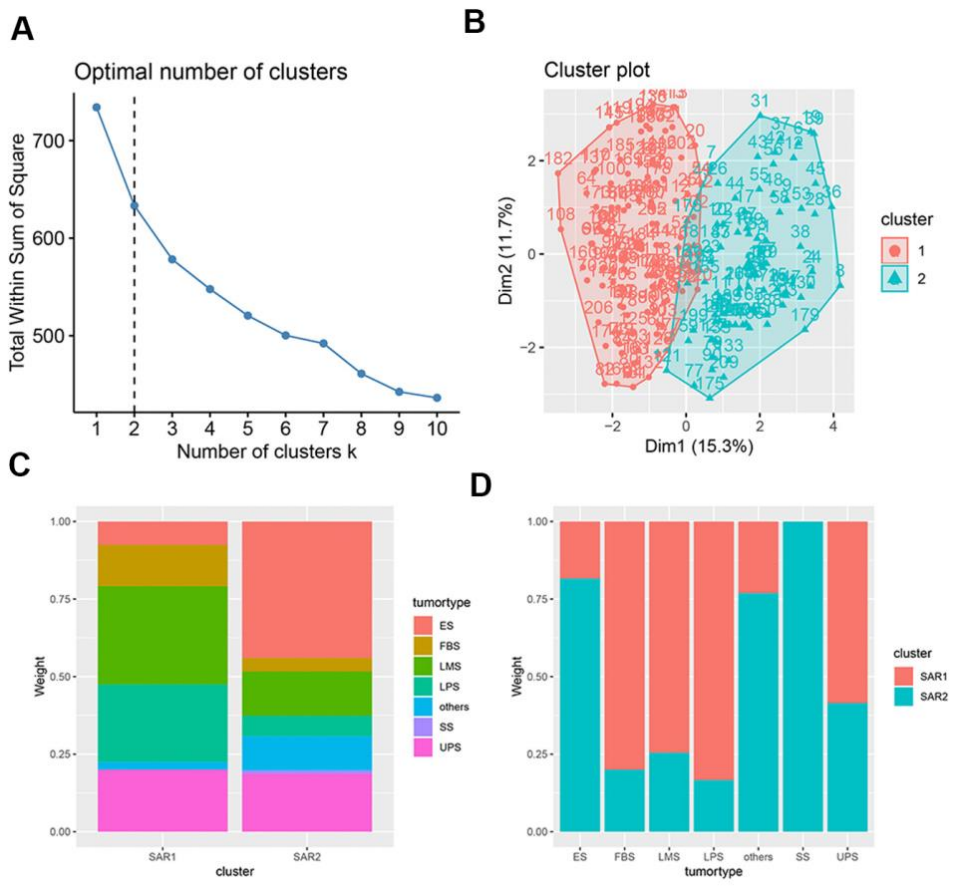
**Characteristics of K-means.** (**A**) The selection of optimal clustering number, the dotted line indicates the chosen number; (**B**) the result of K-means clusters, the results of clustering is shown in two-dimensions, the x axial and y axial represents the characteristics of immune cell infiltration, each dot represents a sample; (**C**) the tumor proportion in SAR1 and SAR2 group; (**D**) the cluster proportion in 7 tumor types.

**Figure 5:**
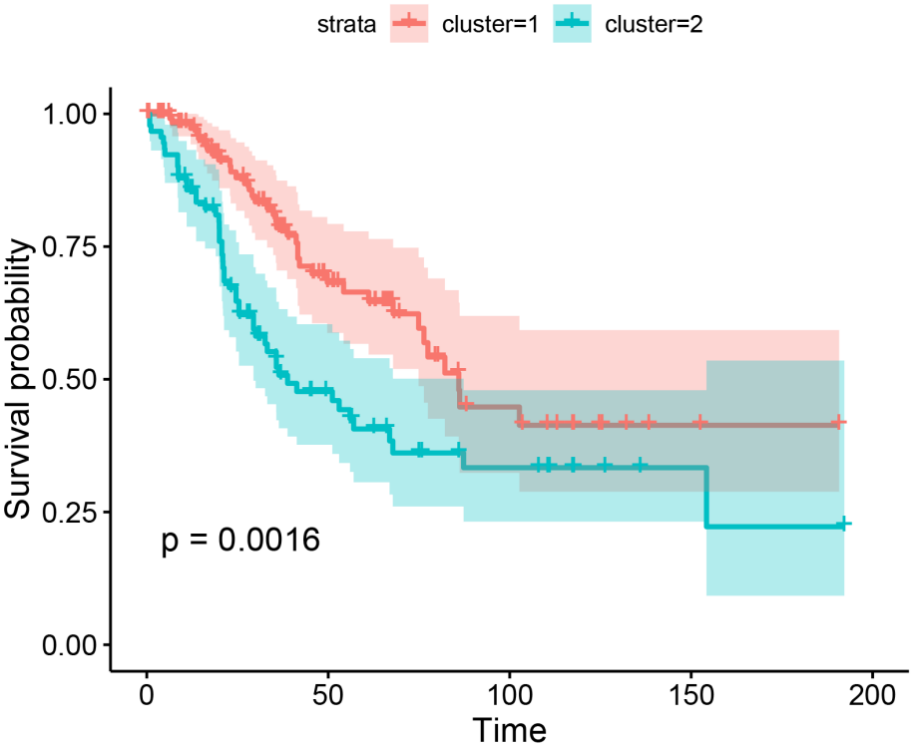
Kaplan-Meier curves for overall survival by the results of K-means.

Sarcoma patients (patients with osteosarcoma were excluded) were regrouped into two groups (SAR1 and SAR2) by the K-means algorithm. The immune cell fractions after re-clustering were shown in Figure 3B. The grouping process is shown in Figure 4A, 4B. Figure 4C, 4D indicate that the proportion of tumor type in SAR1 and SAR2 groups is quite different. SAR1 has a relatively higher proportion of CD8+ T cells, macrophages.M1 and mast cell resting, and a lower proportion of macrophages.M0 than SAR2. More than 70% of Ewing sarcomas are divided into SAR2 groups, while the percentage of fibrosarcoma, leiomyosarcoma, and liposarcoma is less than 30% in this group. After this, we conducted a survival analysis and found a significant difference (p=0.0016). The results were visualized in Figure 5, and the basic characteristics of each group were summarized in Table 1.

### Derivation of the immunoscore

**Figure 6:**
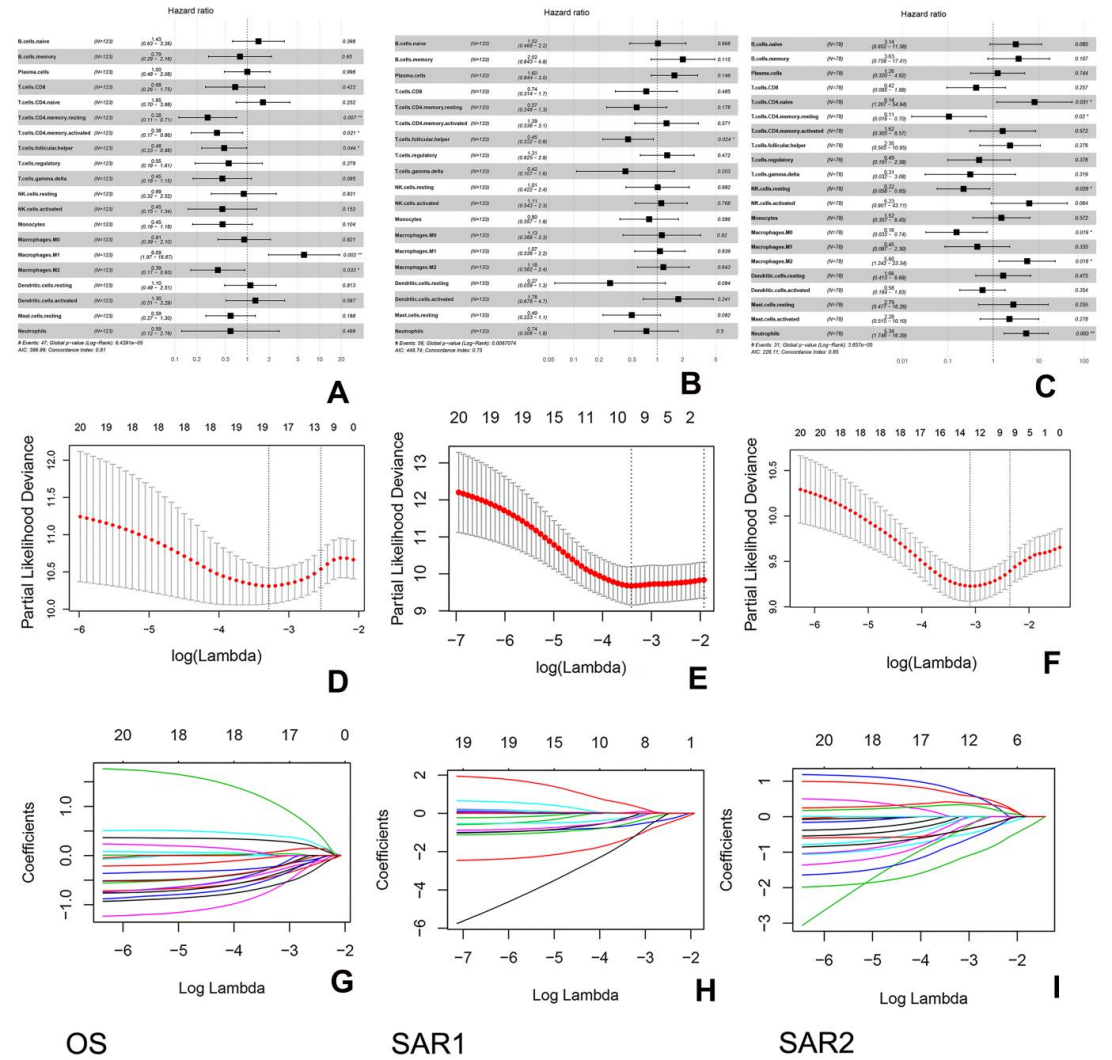
**Construction of the immunoscore in 3 groups.** (**A**–**C**) Forest plots show the association between immune cell subsets and overall survival in 3 groups. The hazard ratios are shown with 95% confidence intervals; (**D**–**F**) Tenfold cross-validation for parameter selection in the LASSO model, the partial likelihood deviance is plotted against log (λ). The subscripted values are the value of log (λ), while the superscripted values are the number of parameters. The partial likelihood with error bars representing standard error is shown. The two dotted vertical lines are drawn at the optimal values of log (λ) (minimum criteria and 1-s.e. criteria); (**G**–**I**) Least absolute shrinkage and selection operator (LASSO) coefficient profiles of immune cell types. These lines represent the coefficient of the corresponding immune cell.

**Figure 7:**
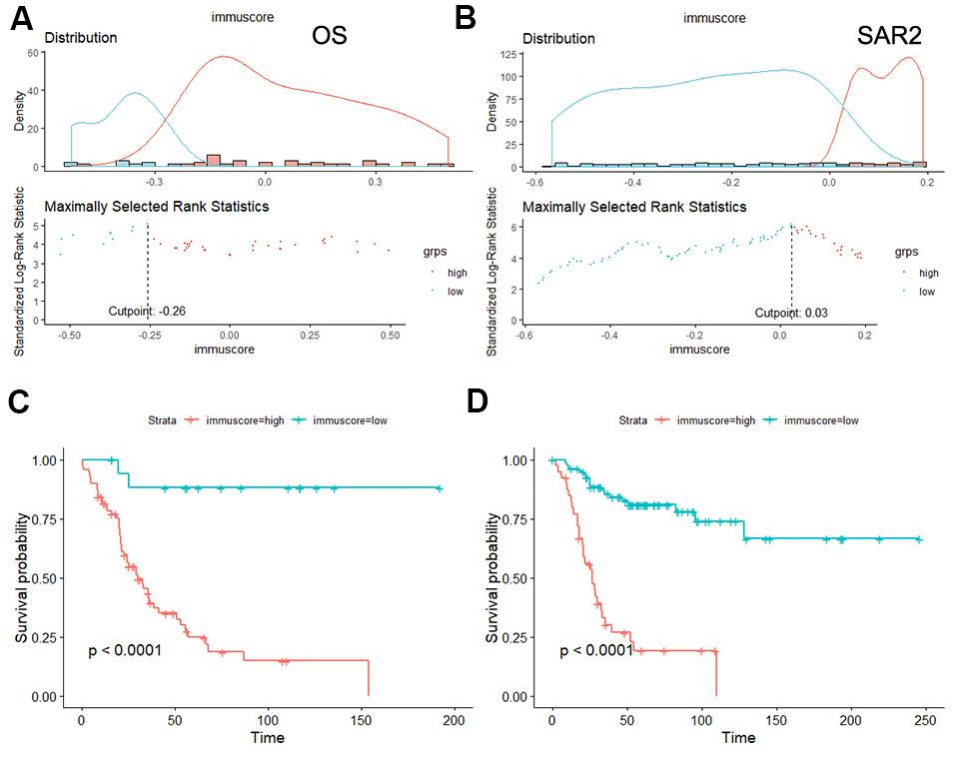
**Survival analysis of the immunoscore in OS and SAR2.** (**A**, **B**) The distribution of immunoscore and the selection of cut-off point, (**A**) OS group, (**B**) SAR2 group; (**C**, **D**) Kaplan-Meier curves for overall survival by immunoscore group.

The survminer R package was used to calculate the optimal cut-off values for a fraction of immune cells. Then, the immune cell fraction level was assigned 0 or 1. The value of 0 indicated the fraction of this type of cell was less than the corresponding cut-off value, and value of 1 otherwise. Figure 6A–6C shows the associations between overall survival time and the immune cell subtypes among 3 groups. The immunoscore model was constructed by LASSO Cox regression analysis (The process is shown in Figure 6D–6I). In our study, the 1-s.e. criteria was used to choose the optimal value of log(λ). Interestingly, the model constructions were only succeeded in 2 groups (OS group and SAR2 group) but failed in the other (SAR1 group) due to the number of parameters was shrink to 1 by the optimal value of log(λ). As shown in Table 2, the model of OS group was estimated by 12 cell fractions, while the model of SAR2 group was predicted through 9 cell types. The formulas for the calculation of immunoscore are also presented. After that, patients were assigned to a high- or low- immunoscore group according to the cut-off value evaluated by the survminer package (0.03 in OS group and -0.26 in SAR2 group). In both groups, patients with higher immunoscores had a relatively poor prognosis (p<0.0001) (seen in Figure 7). The 5-year survival rates were 49.4% (41/83) and 12.5% (5/40) for the low and high immunoscore group respectively in OS. Similarly, the rates were 63.2% (12/19) and 12.3% (9/73) in low and high immunoscore group in SAR2.

**Table 2:** Coefficients of included immune subsets after LASSO regression in OS and SAR2 group.

immunoscore(OS)=0.17358004*B.cells.naive-0.07435504*T.cells.CD8+0.24443641*T.cells.CD4.naive-0.13408067*T.cells.CD4.memory.resting-0.28459064*T.cells.CD4.memory.activated-0.18129293*T.cells.follicular.helper-0.12847892*NK.cells.activated-0.09804615*Monocytes+0.14351879*Macrophages.M0+0.51254662*Macrophages.M1-0.10115331*Macrophages.M2-0.01379196*Neutrophils
immunoscore(SAR2)=0.3745709*B.cells.memory-0.7294329*Plasma.cells+0.2010065*T.cells.CD8-0.1352380*T.cells.regulatory-0.2372486*NK.cells.activated+0.1569576*Macrophages.M2-0.2216186*Dendritic.cells.resting-0.1342080*Mast.cells.activated+0.2947253*Neutrophils

### Nomogram construction

**Figure 8:**
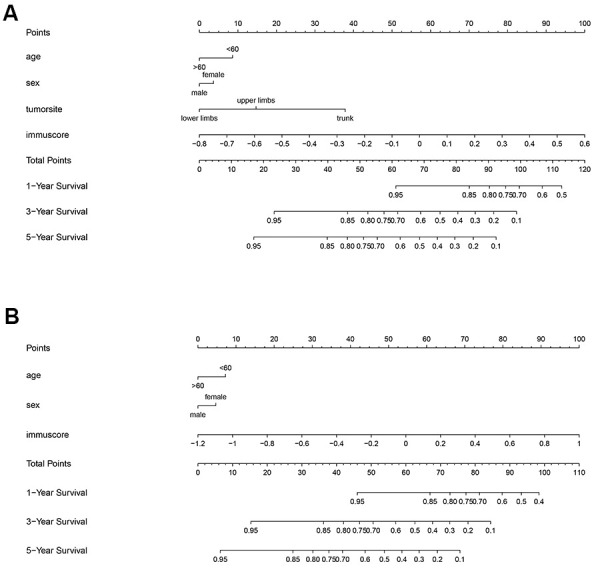
**Nomogram of the 2 groups.** The first axis shows the reference points of included factors. Points of factors are calculated by the corresponding position in the first axis. The corresponding positions of total points in the last 3 axes indicate the 1, 3 and 5 years survival rate. (**A**) OS group, (**B**) SAR2 group.

To predict the probability of overall survival, we constructed nomogram plots to integrated immunoscore and several clinicopathological factors (because clinic information in GEO chips is incomplete, only some of them are included in the construction), shown in Figure 8. In this plot, we could get the total points by simply accumulating each factor’s points through their corresponding position in the first axis. By comparing the total points with reference in the last three axes, we could roughly estimate the overall survival rate at 1, 3, and 5 years. In general, lower points indicate better prognosis. As the figure told, the immunoscore showed ideal predicting effects in both OS and SAR2 groups, and it was an independent factor for overall survival. The prognostic accuracy of the model was assessed by C-index, which was 0.772 in the OS group and 0.722 in the SAR2 group.

### Correlations between immunoscore and clinical characteristics

**Figure 9:**
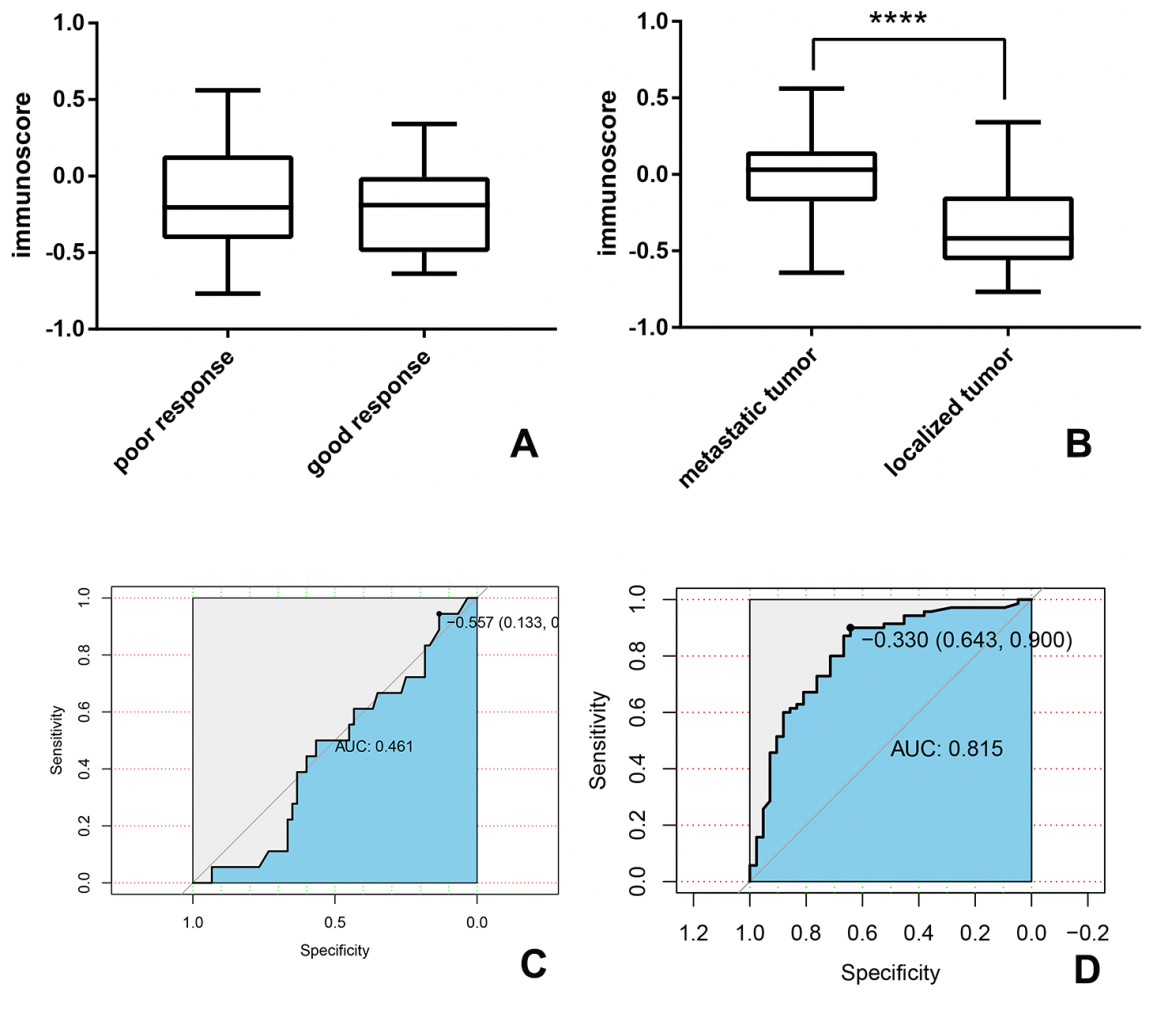
**The association between immunoscore and metastasis and chemotherapy-resistant in the OS group.** (**A**, **B**) the immunoscore of the patient with (**A**) good or poor response to chemotherapy (**B**) localized or metastatic tumor; (**C**, **D**) time-dependent receiver-operating characteristic (ROC) curves present the association between immunoscore and (**C**) chemotherapy-resistant (**D**) metastatic tumor.

The correlations between immunoscore with tumor metastasis and chemotherapy-resistant were further investigated in the OS group. In our study, we found immunoscores varied obviously between patients with localized and metastatic disease (p<0.001), patients with metastatic diseases had higher immunoscores. And as shown in Figure 9, the area under the curve (AUC) was assessed to be 0.815, which further confirmed that correlation was significant. Additionally, patients were divided into two groups: high huvos grade (III/IV) or good response to chemotherapy and low huvos grade (I/II) or poor response to chemotherapy. We found there was no apparent relation between the immunoscore and chemotherapy-resistant.

## DISCUSSION

In our study, we developed a novel classified method and prognostic tool of bone and soft-tissue sarcoma. We found the immune components were diverse in different types of sarcomas. The samples were regrouped into 2 subgroups (SAR1 and SAR2) depend on the immune composition, and a significant difference in survival time between the 2 groups was observed. The immunoscore model was successfully constructed in two subgroups (OS and SAR2). The immunoscore of OS is based on the fractions of 12 immune cells, while the model of SAR2 is based on 9. We proposed the prognostic tool showed a superior ability of survival prediction in both groups, and nomogram plots further validated the results. Patients with higher immunoscore had a worse prognosis, and the higher frequency of metastasis could explain this result. To our knowledge, this is the first study that put forward both the diagnostic and prognostic value of immune cells at the same time in bone and soft-tissue sarcomas.

Sarcomas are rare types of malignancies derived from the mesenchymal lineage, including bone, cartilage, fat, muscle vessels, lymphatics, and nerve. Of note, sarcomas are classified into more than 100 subtypes despite the rarity [22]. Although some aggressive interventions have been developed, patients’ overall survival time was not significantly improved for decades. Outcomes of metastatic disease patients are extremely low. The five-year survival rate is 10-30%. Currently, the most used prognostic system is the Enneking staging system, which identified sarcomas based on three factors: surgical grade (G), surgical site (T), and tumor metastasis (M) [1, 23]. The Enneking staging system was proposed in the 1980s, trying to point out the design of surgical procedures at that time [23], and did not take into account the presence of emerging treatment during the last 30 years.

In recent years, there have been several significant advances in understanding pathogenesis and progression of bone and soft-tissue sarcoma. The tumor micro-environment, comprising various cellular and molecular factors, plays a vital role in the biological behavior of cancer [24]. Tumor-infiltrating immune cells represent the host immune reaction and are considered the leading player in the tumor microenvironment [25, 26]. With these results, immune checkpoint blockade targeting cytotoxic T lymphocyte-associated antigen 4 (CTLA-4) and programmed cell death protein 1 pathway (PD-1/PD-L1) have been investigated and tested in the treatment of sarcoma. However, not as effective as in other tumors, immunotherapies’ responses in bone and soft-tissue sarcoma were controversial [27–29]. The response was associated with the infiltration of immune effector cells [18]. Some specific patterns of tumor-infiltrating lymphocytes (TILs) are associated with improved outcomes in patients with GIST, angiosarcoma, leiomyosarcoma, synovial sarcoma, and undifferentiated pleomorphic sarcoma [13, 30–32].

Additionally, tumor-associated macrophages (TAMs) also had an essential role in tumor invasion and metastasis [11, 33, 34]. They can release numerous factors that could influence tumor cells’ biological behavior and tumor stroma [35]. Besides, recent evidence indicated that the immune feature varied across tumor subtypes [9, 36], which means these features could be used as diagnostic biomarkers. Given that we noted both the prognostic and diagnostic value of immune cells, we hypothesized that immune composition could be an efficient categorization tool for bone and soft-tissue sarcomas.

Previously, immunohistochemistry was the most common strategy for studying immune cell heterogeneity. However, this method has some technical restrictions because it heavily relies on `the sufficient size of biopsy specimens and a limited repertoire of phenotypic markers. So these studies were always limited by either rare cell types or insufficient sample size. In contrast, CIBERSORT is a newly proposed computational algorithm that can estimate immune cell types’ fractions based on gene expression profiling series. It was possible to generate an expanded view of the immune response at the cellular level by applying an analytical method to public genomic data.

CIBERSORT thoroughly analyzed the immune fractions of 334 included samples. According to the results, soft-tissue sarcoma patients were further divided into 2 subgroups (osteosarcoma was excluded in this classification due to the bone-immune system’s existence, which is unique in bone organ [37], could affect the consistency of results). Significant differences in the immune composition were detected, and both groups had their specific immune infiltrating patterns.

Visible separation of overall survival curves was found between patients in SAR1 and SAR2 groups. Patients in the SAR1 group have a favorable outcome and a higher proportion of three immune cells: CD8 ^+^ T cells, macrophage cell (M1), and mast cells. Previous studies indicated an increased level of these cells contributes to a good prognosis in patients with different malignancies. CD8 ^+^ T cells, holding the capacity to kill tumor cells directly, was considered to be associated with improved survival across many types of cancers [10, 38–40]. As for M1 macrophages, they could produce some effector molecules (reactive oxygen intermediates, reactive nitrogen intermediates, and TNFs) to limit tumor growth and recruit cytotoxic T cells. Also, the tumoricidal capacity of M1 macrophages was well studied. Several studies indicated that the M1 macrophage cells’ ratio was significantly associated with improved prognosis in various tumor types [41–44]. Mast cell, an essential component of the tumor microenvironment, can be part of innate and acquired immune responses and contribute to many pathophysiological conditions [45]. However, the specific mechanism of mast cell remained unclear. Producing a broad repertoire of cytokines, mast cells have been shown both pro-and antitumor effects [46–49]. Previously, mast cells were mostly found at the tumor-bone interface as a biomarker for osteolysis [50], so the bone destruction of sarcoma could be another possible reason for the extent of infiltration.

In addition, we have noticed that the age structure of SAR1 and SAR2 groups were quite different (<18y: 44.87% vs. 3.76%). On the one hand, this was because of the age distribution of sarcomas. Ewing sarcoma, accounting for 50% in SAR1, is the second most common sarcoma type among adolescents [51]. While leiomyosarcoma and liposarcoma, taking the leading position in SAR2 group, are the most frequent histypes seen in adults [52, 53]. On the other hand, this distribution could also be an explanation of the difference in survival time. A previous study has indicated that the survival declined with advancing age in most sarcoma histologies [53].

Another interesting finding is that sarcomas of different pathological types may have similar immune patterns, but the pattern can be different within one sarcoma type. In our opinion, this might because the interaction between the host immune system and tumor cells changed during tumor development and varied in different types of tumors, which can also be seen in some experimental analysis [50, 54]. Genetically, soft tissue is often divided into two categories: “simple” and “complex” through the oncogenic alterations and the number of mutations. Liposarcoma, synovial cell sarcoma, and gastrointestinal stromal tumor were considered to be “the simple tumor,” while undifferentiated pleomorphic and leiomyosarcoma were characterized as “the complex tumor.” Nevertheless, this grouping method was not successful every time. Researchers have found that patients with conventional “simple” sarcoma can be clustered to the complex group some time [9]. Similar results were also obtained by Abeshouse et al., who re-clustered 206 sarcoma samples to 3 subsets by immune infiltration. They found the immune signatures could be largely different within the same tumor type [55].

After the regrouping of soft tissue sarcoma, we further proposed a novel prognostic tool. The model was successfully constructed in the OS group and SAR2 group, based on 12 and 9 immune cells, respectively. Survival curve analysis revealed that high immunoscores was associated with poor overall survival time. However, effective immune cell types were divergent between the two groups. Therefore, we speculate the same immune cells might have different prognostic roles in other groups. This assumption is in line with past researches and can explain some discordances in previous studies. For instance, an article pointed out infiltration of CD8+ lymphocytes were associated with favorable outcome in patients with synovial sarcoma [13], which was confirmed by a meta-analysis [38]. Other researchers, however, thought the association was not significant in their samples [19].

Several studies have given rise to the idea that the infiltration of the immune cells was associated with chemotherapy-sensitivity [39, 56] and metastasis [57, 58] in multiple diseases. To confirm this hypothesis in sarcoma patients, we compared these two possible factors subsequently. In our results, metastatic tumors are likely to have a higher score than the localized disease (p<0.001), while the difference between chemotherapy-sensitive and chemotherapy-resistant groups was not statistically significant (p>0.05). Considering all the included samples are derived from the primary tumor, we hypothesized the immunoscore could use to predict the risk of metastasis. The receiver operating characteristic (ROC) curve were conducted then and the area under the curve (AUC) is 0.815. Metastases are quite common in sarcoma patients and the presence of metastatic lesions has conventionally implied an incurable state of disease. If postoperative pathology of primary tumor is capable of predicting mobilization, patients will more likely to receive timely confirmation and treatment.

Unexpectedly, we failed to construct an appropriate model for the SAR1 group. In our view, a likely interpretation is that immune components have a paradoxical impact on the tumor process. Cytokines with tumorigenic ability can also function as a tumor suppressor in some cases. It depends on when they are recruited into this microenvironment [54]. For example, MyD88 and IL-1β have protumor activities at an early stage [59] and antitumor activities at a later stage [60] in mouse tumor models. Samples included in our unsupervised clustering comprised various tumor stages and quantity, density, and prognostic significance of these immune components changed during this process. The interaction of host immunity and tumor formation is usually in a subtle dynamic balance, changing almost every time. We assume the specific immune pattern in SAR1 group may exist more than once in this process. So the combined results of different stages samples could be confusing to some extent.

The immunoscore system is a novel tool designed to re-cluster and predicts bone and soft tissue sarcoma’s survival. In recent years, several models based on gene expression profiles and immunoscore have indicated that the immune contexture could be a statistically important parameter for the prognosis and classification in patients with different types of malignancies [17, 61–64]. Besides, recent studies have introduced the immunoscore to the current AJCC/UICC TNM classification of colon cancer, designated TNM-I (TNM-Immune) [61, 65]. However, the analysis of immunoscore in bone and soft tissue sarcoma is scarce. The majority of previous studies only focused on the infiltrating of two or three immune components, such as PD-L1, CD8+ T cell, CD4+ T cell, NK cell, and macrophages [9, 13, 66].

In contrast to these studies, the candidate immune cells used in our study included 22 types. The immunoscore was estimated based on high-throughput gene expression data from 3 public databases by using newly developed algorithm CIBERSORT. It allowed a broader and precise view of the immune interaction, with a relatively larger patient cohort than previous studies. In this view, our model will be helpful in genetic counseling services by evaluating the recurrence/metastasis risk of sarcoma patients [67, 68].

This study also has some limitations. Our study was based on public data sets, so it was impossible to obtain all the needed information for every patient. In addition, some of the available data sets only provided insufficient expression data so that the final included sample size was significantly shrinking. It is the most important reason we could not further divide our samples into training and validation cohorts. Secondly, the gene expression profiles used here were all derived from the center of the mass (some of this information was not available), so we cannot take the immune cells’ location into account when constructing the immunoscore model. Thirdly, all patients selected in this study retrospectively, the inherent limitation of retrospective design should not be ignored.

## MATERIALS AND METHODS

### Searching for eligible datasets

To obtain soft tissue sarcoma gene expression data, systematic searches were conducted by two investigators individually. The microarray data were collected from three online databases, including Gene Expression Omnibus (GEO) database (https://www.ncbi.nlm.nih.gov/geo/), The Cancer Genome Atlas (TCGA) database (https://cancergenome.nih.gov/) and The Therapeutically Applicable Research to Generate Effective Treatment (TARGET) program (https://ocg.cancer.gov/programs/target). Search terms in the GEO database were as follows: ((survival or prognosis or prognostic or outcome or death or relapse or recurrence) and sarcoma) and ‘Homo sapiens.’ The inclusion and exclusion criteria of the series collection are shown in Figure 1.

### Data processing

The “GEOquery” package of R software (x64, version 3.5.0, R Project for Statistical Computing, Vienna, Austria) was applied to download GEO microarrays. Raw microarray data (.CEL files) were normalized by the robust multiarray averaging method with “affy” and “simpleaffy” packages. If the “CEL” file can not be acquired, the normalized matrix files were downloaded instead. Analyzed TCGA data including mRNA expression and clinical files were obtained from the abovementioned website. Besides, open-access data of osteosarcoma in TARGET data matrix was downloaded through the recommended way (https://ocg.cancer.gov/programs/target/using-target-data). For all these files, gene IDs were converted to the official gene symbol by “AnnotationDbi” and “org.Hs.eg.db” packages. Before the next step analysis, data processing was already done by “caret” package, chips were excluded because of obvious collinearity and near zero.

### Collection of clinical data

Information was collected on age, sex, tumor type, tumor size, tumor site, tumor stage, and overall survival time (OVS). Among these factors, sex, tumor type (Ewing sarcoma, fibrosarcoma, leiomyosarcoma, liposarcoma, osteosarcoma, rhabdomyosarcoma, synovial sarcoma, undifferentiated pleomorphic sarcoma, and others), tumor size (>5cm or not), tumor site (lower limbs, upper limbs, trunk, and others), tumor stage (low grade and high grade) were considered as categorical variables while age was regarded as a continuous variable. The detailed information was shown in Table 1.

### CIBERSORT estimation

After the abovementioned data processing, we uploaded gene expression data to the CIBERSORT website ((http://cibersort.stanford.edu/). The algorithm was run with LM22 signature and 1000 permutations [21]. The LM22 signature consists of 547 genes that can accurately distinguish 22 human hematopoietic cell phenotypes, including T cell types, B cells, plasma cells, NK cells, and myeloid subsets. For the accuracy of results, only those samples with a p-value of less than 0.05 in the final CIBERSORT output were considered to be included [17].

### Statistical analysis

Statistical analyses were performed using R software (version 3.4.0) and GraphPad Prism 7 software. P value< 0.05 was considered to be statistically significant. The survival curves were constructed by the Kaplan-Meier method through “survival” and “survminer” package, and results were compared by means of the log-rank test. Comparison among groups was conducted using the independent t-test for continuous variables and X2 test for categorical variables. K-means algorithm was applied to reclassify categories of sarcoma by considering LM 22 signature. To estimate the optimal number of clusters, “wss” method (total within the sum of square) was employed, and the results were visualized by “factoextra” package. For reducing the dimensionality of data, the least absolute shrinkage and selection operator (LASSO) analysis was utilized by “glment” package. The optimal value of λ (penalty parameter) was evaluated by ten-fold cross-validations [69]. The predictive value of immunoscore was assessed by uni- and multivariable cox regression using ovs (overall survival time) as outcome variable and age, sex, tumor type, tumor size, tumor site, and immunoscore as covariates. Next, the nomogram was constructed by “rms” package and the accuracy of the model was assessed by the concordance index (C-index). When the C-index is equal to 0.5, indicating that the model has no predictive function, when the index is equal to 1, the result predicted is entirely consistent with the actual conditions. Besides, patients were further classified according to their clinical characteristics such as metastasis and response to preoperative chemotherapy. The t-test conducted a comparison of immunoscore among groups, and the results were visualized by Graphpad Prism 7.0 software. Accuracy of this immunoscore model was evaluated by the receiver operating characteristic (ROC) curve calculated through “pROC” package, and the area under the curve (AUC), were used to evaluate the predictive value of immunoscore.

### Contribution to the field statement

This article provided a novel sight in the classification and prognosis of bone and soft tissue sarcoma by the immune composition. RNA-seq data from three databases including 334 samples, were recruited in this analysis. The CIBERSORT website evaluated each patient's immune components as our results indicated that the overall survival is strongly associated with the type and amount of immune cell infiltration. Besides, we further divided the soft tissue sarcoma samples into 2 subgroups by the K-means algorithm and found an obvious survival difference between them. Then, we constructed a prognostic model by lasso regression and calculated the immunoscore, which showed a great prognostic value. To our knowledge, this is the first study to introduce immune infiltration into the classification and prognosis of bone and soft tissue sarcoma. We hope our results can provide a new thought for future studies.

### Data availability statements

The data analyzed for this study can be found in the TCGA (https://cancergenome.nih.gov/), GEO (https://www.ncbi.nlm.nih.gov/geo/) and TARGET (https://ocg.cancer.gov/programs/target) databases.
